# Small changes in lung function in runners with marathon‐induced interstitial lung edema

**DOI:** 10.14814/phy2.12056

**Published:** 2014-06-27

**Authors:** Gerald S. Zavorsky, Eric N.C. Milne, Federico Lavorini, Joseph P. Rienzi, Paul T. Cutrufello, Sridhar S. Kumar, Massimo Pistolesi

**Affiliations:** 1 Department of Health and Sport Sciences, University of Louisville, Louisville, 40292, Kentucky; 2 Department of Physiology and Biophysics, University of Louisville, Louisville, 40292, Kentucky; 3 Department of Radiological Sciences, University of California – Irvine, Irvine, 92697, California; 4 Section of Respiratory Medicine, Department of Experimental and Clinical Medicine, University of Florence, Florence, 50134, Italy; 5 Department of Radiology, Regional Hospital of Scranton, Scranton, 18510, Pennsylvania; 6 Department of Exercise Science and Sport, The University of Scranton, Scranton, 18510, Pennsylvania; 7 Great Valley Cardiology, Scranton, 18510, Pennsylvania

**Keywords:** Endurance, exercise, lung fluid, lung function, pulmonary, water

## Abstract

The purpose of this study was to assess lung function in runners with marathon‐induced lung edema. Thirty‐six (24 males) healthy subjects, 34 (SD 9) years old, body mass index 23.7 (2.6) kg/m^2^ had posterior/anterior (PA) radiographs taken 1 day before and 21 (6) minutes post marathon finish. Pulmonary function was performed 1–3 weeks before and 73 (27) minutes post finish. The PA radiographs were viewed together, as a set, and evaluated by two experienced readers separately who were blinded as to time the images were obtained. Radiographs were scored for edema based on four different radiological characteristics such that the summed scores for any runner could range from 0 (no edema) to a maximum of 8 (severe interstitial edema). Overall, the mean edema score increased significantly from 0.2 to 1.0 units (*P *<**0.01), and from 0.0 to 2.9 units post exercise in the six subjects that were edema positive (*P = *0.03). Despite a 2% decrease in forced vital capacity (FVC,* P *=**0.024) and a 12% decrease in alveolar‐membrane diffusing capacity for carbon monoxide (DmCO,* P *=**0.01), there was no relation between the change in the edema score and the change in DmCO or FVC. In conclusion, (1) mild pulmonary edema occurs in at least 17% of subjects and that changes in pulmonary function cannot predict the occurrence or severity of edema, (2) lung edema is of minimal physiological significance as marathon performance is unaffected, exercise‐induced arterial hypoxemia is unlikely, and postexercise pulmonary function changes are mild.

## Abstract (precis)

This study assessed lung function in runners with marathon‐induced interstitial lung edema. Pulmonary function tests and chest radiographs were obtained pre‐ and post marathon finish. Seventeen percent of subjects developed edema but the edema was of minimal physiological importance.

## Introduction

Marathon running is a popular event. There were ~541,000 marathon finishers in the United States in 2013 (Running USA Annual Marathon Report). Due to its popularity, there has been scientific research interest accruing over the past few decades on the physiology of marathoners. Recently, it has been demonstrated that the lung may be a limitation to marathon performance by demonstrating that those with higher pulmonary diffusing capacities at rest have faster finishing times (Lavin et al. 2012). Furthermore, as marathon running reduces pulmonary diffusing capacity postexercise (Manier et al. 1991), then it's likely that this reduction would be associated with increases in extravascular lung water leading to mild interstitial pulmonary edema.

The development of pulmonary edema with intense, upright, sea level exercise is controversial (Hopkins 2010b,b; Sheel and McKenzie 2010a,b), and some of the controversy is due to varied imaging techniques to detect edema. However, chest radiography has been demonstrated to be the most practical method to detect and quantify pulmonary edema for clinical purposes (Staub 1986; Milne and Pistolesi 1993; Ware and Matthay 2005) since the level of accuracy of the radiological quantification of edema has been determined rigorously using a modified double indicator dilution technique (Pistolesi and Giuntini 1978).

Case reports on three subjects were published in 1979 and 1987 demonstrating that pulmonary edema could be triggered from marathon or ultramarathon running (McKechnie et al. 1979; Young et al. 1987) but those cases of alveolar pulmonary edema are rare. Our study was the first that specifically sought to examine the incidence of pulmonary edema (alveolar or interstitial) triggered by marathon running (Zavorsky et al. 2014). The results demonstrated that several runners developed mild interstitial pulmonary edema triggered by marathon running and that females showed a higher likelihood of developing mild interstitial pulmonary edema compared to men (Zavorsky et al. 2014). Thus, the purpose of this study was to include documentation of clinical signs and symptoms of pulmonary edema, in addition to pulmonary function data for association with radiological edema scores in marathon runners. It was hypothesized that there would be a relation between the clinical manifestation of pulmonary edema and radiological scores of edema.

## Methods

### Study participants

Forty subjects were recruited, but 36 (24 males, 34 [SD 9] years, body mass index = 23.7 [2.6] kg/m^2^) completed the study (four were lost to attrition). Times from the 2012 Steamtown marathon (Scranton, PA) ranged from 149 to 295 min. The inclusion criteria were as follows: any male or female ≤55 years of age, with the ability to complete the marathon under 5:00 h, and with no symptoms or known presence of heart disease. Five of the 36 subjects were also participants of the 2014 study (Zavorsky et al. 2014). The exclusion criteria were as follows: signs or symptoms of cardiopulmonary disease or obstructive/restrictive airways determined by spirometry. The study was approved by the Institutional Review Boards of Marywood University and The University of Scranton. Informed consent was obtained from each subject prior to study commencement.

### Chest radiographic images

Each subject had one posterior/anterior (PA) radiograph taken 1 day before the race and another 21 (6) minutes post finish. All radiographic imaging was performed using a portable X‐ray machine (Model SR‐115, Source Ray Inc., Ronkonkoma, NY) and Digital Radiology ViZion DR imaging Software/flat panel detectors (Viztek Inc., Garner, NC). The X‐ray tube‐to‐patient distance was six feet. Radiographic exposure was individualized using 90 to 125 kVp and 2.4 mAs (6 ms) to 9.8 mAs (24 ms) exposure time, depending on the subject's anthropometric characteristics and using the lower kV levels to obtain optimum lung contrast. The image was obtained at total lung capacity. Each radiograph provided an effective radiation dose ranging from 0.02 to 0.12 mSv (Wall and Hart 1997). Thus, the maximum total exposure per subject was 0.24 mSv (2 × 0.12) for the full study.

Each X‐ray set was randomly coded and two readers were blinded as to the time each radiograph was taken. The two readers have varying levels of expertise in reading chest films: E.N.C.M. (chest radiologist) has 40 years of experience in physiological interpretation of the chest radiograph, and specifically in the X‐ray quantification of pulmonary edema. He has previously published a textbook on reading the chest radiograph (Milne and Pistolesi 1993). The second reader, F.L. (pulmonologist) was a research fellow of M.P. and has 10 years of experience.

Each reader was given the same set of instructions for interpreting the films and evaluated the films separately from the other readers. The readers were aware of the general study design but were not informed of the time at which the radiographs were taken or of the performance of the runner or of the interpretation given by other physicians. Each reader analyzed all the pre‐ and post exercise radiographs from one subject concurrently, in randomized order. The reading of each radiograph, for a given time point, was completed before the reader proceeded with the next radiograph.

Posterior/anterior (PA) chest radiographs were obtained 1 day before the marathon and 21 (6) minutes post finish. Films were analyzed for evidence of pulmonary edema with the use of previously extensively documented criteria (Pistolesi and Giuntini 1978; Milne 1985; Gallagher et al. 1988; Miniati et al. 1988; Milne and Pistolesi 1993; Anholm et al. 1999; Zavorsky et al. 2006). The criteria were based on the presence or absence of each of the following four findings: (A) loss of sharp definition of pulmonary vascular markings; (B) hilar blurring; (C) peribronchial and perivascular cuffing; and (D) obscuration of the smallest peripheral vessels. The first three radiographic findings were graded on a 3‐point scale: 0 if the finding was absent, 1 if the finding was minimally present, and 2 if there was a definite radiographic presence. For the fourth finding, the items were scored as 0 for normal visualization, 1 for partially obscured, and 2 for completely obscured. An edema score was then calculated by summing the score for each of the four radiographic characteristics. The scores for all 36 runners from the two readers were then added and averaged to give the mean overall edema score. Interstitial lung edema was scored as “none” (a total score of equal to or less than 2.0), “mild” (a total score from 2.1 to 3), “moderate” (a total score from 3.1 to 4), and “severe” [total score of 4.1 to a maximum of 8.0 (see Zavorsky et al. (2014)).

### Pulmonary function testing

All lung function tests were done by G.S.Z. 1–2 weeks before the marathon and 73 (SD 27) minutes post finish. Spirometry was measured according to ATS/ERS standardization of spirometry guidelines (Miller et al. 2005). Forced vital capacity (FVC), forced expiratory volume in 1 sec (FEV_1_), forced expiratory flow rate over the middle half of expiration (FEF_25–75_), and peak expiratory flow rate (PEF) were measured as part of the spirometry battery. The subjects' values were compared against reference equations (Hankinson et al. 1999). Pulmonary diffusing capacity for nitric oxide (DLNO) and carbon monoxide (DLCO) were also measured according to the methods described elsewhere (Zavorsky et al. 2008), and subjects' values were also compared against reference equations (Zavorsky et al. 2008). Pulmonary capillary blood volume (Vc) was determined based on the following: Alveolar Po_2_ (P_A_O_2_) = 100 mmHg (Zavorsky et al. 2008), the blood transfer conductance for NO (*θ*NO) = 4.5 mL/min/mmHg/mL (Carlsen and Comroe 1958; Borland et al. 2014), the blood transfer conductance for CO (*θ*CO) = 0.584 mL/min/mmHg/mL when male hemoglobin concentration = 14.6 g/dL, and 0.537 mL/min/mmHg/mL when female hemoglobin concentration = 13.4 g/dL). This was estimated on the blood transfer conductance equation by Forster (1987): 1/*θ*CO = (1.3 + 0.0041∙P_A_O_2_) · (14.6/subject's Hb). Furthermore, the alveolar‐membrane diffusing capacity for carbon monoxide (DmCO) was calculated as the alveolar‐membrane diffusing capacity for nitric oxide (DmNO) divided by 1.97. Thus, DLNO < DmNO (Borland et al. 2010, 2014; Zavorsky 2010). The ratio of DLNO to DLCO was assumed to be an adequate surrogate for the DmCO to Vc ratio (Hughes and van der Lee 2013).

### Clinical signs and symptoms of pulmonary edema

The signs/symptoms of pulmonary edema were evaluated by S.S.K. (M.D., F.A.C.C.) 1 day before the race and 16 (7) minutes post finish. The following were evaluated as a “yes” or “no” by S.S.K.: Bilateral crackles (end‐inspiratory crackles), cough, productive of frothy or blood‐tinged sputum, third heart sound (S_3_ gallop rhythm), and raised jugular venous pressure as evidenced by jugular venous distension. Subjects were asked to report their breathlessness by using the modified Borg scale for perceived dyspnea. Also, postexercise heart rates, blood pressure, and a measurement of arterial oxyhemoglobin saturation via pulse oximetry were also recorded.

### Statistical analyses

Anthropometric characteristics between males and females were compared using independent sample *t*‐tests. Mean values of lung function variables, arterial oxyhemoglobin saturation, heart rate, and blood pressure obtained at baseline and after completion of marathon were compared with paired samples *t*‐tests; intensity of dyspnea (Borg scale) between pre‐ and post finish was compared using a Wilcoxon signed ranks test. Other clinical signs and symptoms of pulmonary edema (bilateral crackles, cough productive of frothy sputum, S_3_ gallop rhythm, and jugular venous distension) pre‐ to post finish were compared using McNemar's test, which is a test to compare paired proportions. Radiographic characteristics before and after exercise for each reader were compared using a Wilcoxon‐signed ranked test. This test compared whether the median edema scores (from 0 to 8, ordinal data) among the pre‐ and post measurement points for all 36 finishers significantly increased. The level of interobserver agreement on the quantification of pulmonary interstitial edema (the scores) was obtained from the weighted *kappa* statistical test (Kundel and Polansky 2003; Jakobsson and Westergren 2005), and the average‐weighted *kappa* coefficient was reported (Kundel and Polansky 2003). To see if there was a mean difference in the changes in the edema score from pre to post exercise between the two readers, a Mann–Whitney *U*‐test was used. Forward binary logistic regression was conducted to determine which independent variables (marathon finishing time, age, and sex) were predictors of developing mild interstitial pulmonary edema (yes or no) from marathon running. A postexercise edema score of ≥2.1 units was considered significant for interstitial lung edema (Zavorsky et al. 2014). The data were analyzed by a statistical software package (SPSS Version 20.0, IBM SPSS Statistics Inc., Chicago, IL). Statistical significance was declared when *P *<**0.05 unless otherwise noted.

## Results

**Figure 1.**
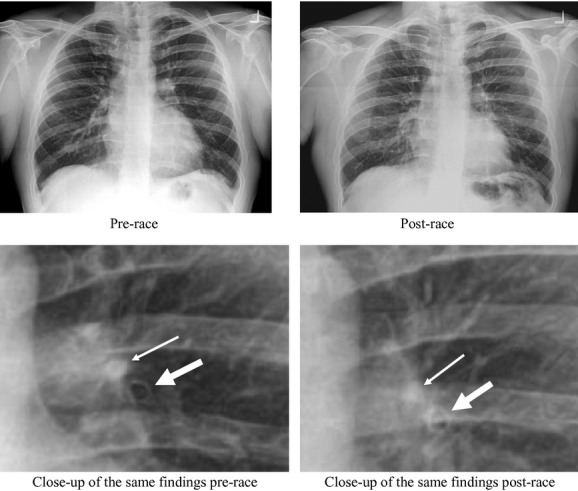
Evidence of postmarathon increase in peribronchial (thicker arrows, bottom) and perivascular (thinner arrows, top) cuffing. Subject was male, 32 years old, BMI = 27.7 kg/min^2^. He completed the marathon in 4 h 25 min. The postexercise radiograph was obtained 20 min post marathon finish.

Forty runners (Table 1) completed the 2012 Steamtown Marathon (which began at 475 m above sea level in Forest City, PA, and finished at 229 m above sea level in Scranton, PA), under 5 h (300 min), ranging from 2:29 to 4:55, with an average (SD) finishing time of 3:47 (SD = 37 min). The ambient temperature increased from about 8°C (78% humidity) at the 8:00 am start to 11°C (78% humidity) at the finish line by 1:00 pm. We were able to obtain prepost radiographs from 36 of the 40 subjects recruited. Lung function, as a whole, was normal in these subjects (Table 2). The preexercise edema scores averaged by two the readers ranged from 0 to 1.5. Both readers showed that the edema score from pre to post finish significantly increased demonstrating a fivefold increase compared prerace (*P *<**0.01, Table 3). Overall weighted kappa was calculated to be 0.57 (95% CI = 0.38 to 0.76) indicating agreement among observers of moderate degree for the quantification of edema. Six subjects (17%) had a postexercise edema score that ranged between 2.5 and 3.5 units out of a maximum score of 8 (mean postexercise edema score = 2.9 units, *P = *0.03 compared to prescores) when averaged between the two readers. These six subjects also had a mean change in the edema score that was +2.9 units greater compared to preexercise. Figure 1 demonstrates a subject with peribronchial and perivascular cuffing obtained 20‐min post marathon finish.

**Table 1.** Anthropometric characteristics.

	Males (*n *=**24)	Females (*n *=**12)	Total (*n *=**36)
Age (years)	36 (8) 19–52	32 (10) 21–48	34 (9) 19–52
Weight at start (kg)	75.7 (8.0) 62.6–86.6	56.5 (4.8)^1^ 49.0–64.3	68.1 (11.5) 49.0–86.6
Height (cm)	175 (7) 163–188	161 (6)^1^ 152–171	170 (9) 152–188
BMI (kg/m^2^)	24.7 (2.3) 20.1–28.5	21.9 (2.1)^1^ 18.4–26.4	23.7 (2.6) 18.4–28.5
BSA (m^2^)	1.91 (0.13) 1.69–2.11	1.59 (0.08)^1^ 1.46–1.70	1.81 (0.19) 1.46–2.11
Percent body fat (%)	15.1 (4.9) 7.4–25.8	23.8 (4.8)^1^ 14.8–33.3	18.0 (6.4) 7.4–33.3

^1^ Indicates significant difference relative to male subjects (*P *<**0.01). Percent body fat was obtained by Dual X‐Ray Absorptiometry.

**Table 2.** Lung function variables (*n *=**36).

	Mean (SD) Range	Percent of predicted value
FVC (L)^1^	5.05 (0.97) 3.18–6.45	108 (11) 82–140
FEV1 (L)^1^	4.02 (0.72) 2.70–5.35	106 (11) 76–125
FEV1/FVC (L)^1^	0.80 (0.6) 0.65–0.90	97 (6) 82–106
PEF (L/s)^1^	9.75 (2.00) 6.00–13.47	110 (15) 78–136
FEF25–75 (L/s)^1^	4.96 (1.04) 3.04–6.86	130 (26) 75–192
DLCO (mL/min/mmHg)^1^	35.0 (7.4) 20.6–50.0	114 (18) 76–166
DLCO/VA (mL/min/mmHg/L)	5.1 (0.7) 3.9–6.9	–
DLCO/BSA (mL/min/mmHg/m^2^)	19.2 (2.8) 13.2–25.3	–
DLNO (mL/min/mmHg)^1^	173 (39) 107–265	108 (18) 78–155
DLNO/VA (mL/min/mmHg/L)	25.4 (3.1) 21.2–32.9	–
DLNO/BSA (mL/min/mmHg/m^2^)	95.3 (15.0) 68.7–129.4	–
DLNO/DLCO ratio	4.96 (0.35) 4.24–5.96	–
DmCO/Vc ratio	2.39 (0.65) 1.38–4.72	–
DmCO (mL/min/mmHg)	183 (61) 102–411	–
Vc (mL)	77 (14) 47–109	–

^1^ Denotes significant difference (*P *<**0.05) relative to predicted value.

**Table 3.** Overall scores for interstitial pulmonary edema (*n *=**36).

Time	Reader #1 E.N.C.M.	Reader #2 F.L.	Overall
Pre	0.1 (0.1)	0.3 (0.1)	0.2 (0.1)
Post	1.3 (0.3)	0.8 (0.2)	1.0 (0.2)
Change	+1.2 (0.3)	+0.5 (0.2)	+0.9 (0.2)
*P*‐value	0.001	0.035	0.000

Mean (SE) The scores range from 0 (no edema) to 8 (severe interstitial edema). Both readers showed significant increase in the edema score from pre to post marathon. There was no difference in the mean changes between the two readers (*P *=**0.411). In the six subjects who were edema positive, the preexercise edema score was 0.0 (0.0), and the postexercise edema score was 2.9 (0.2) (*P = *0.03, compared to preexercise scores).

Only 25 subjects stayed for their lung function assessment after their postexercise radiographs were obtained. Overall, there were small but significant decreases in FVC (2%) and DLNO indexed to alveolar volume (VA) post exercise (6%, Table 4). No changes in DLCO or Vc were evident. There was a ~12% decrease in DmCO and the DmCO/Vc ratio (Table 4). The decreases in FVC, DLNO and DLNO/VA, or DmCO were not related to the increase in the edema score. Two of the six edema‐positive subjects did not stay for lung function assessment after their postexercise radiographs were obtained. Thus, we only have postexercise lung function data in four of the six edema‐positive subjects. In these four edema‐positive subjects, DmCO decreased by 27 (32) mL/min/mmHg, DLNO decreased by 7 (20) mL/min/mmHg, and FEV_1_ decreased by 100 (50) mL. FVC and FEF_25–75_ were not affected in these four positive edema subjects.

**Table 4.** Change in lung function variables post marathon compared to baseline.

	Mean Δ (SD) [95% CI]	*P*‐value
FVC (L)	−0.10 (0.21) [−0.19, −0.01]	0.024^1^
FEV_1_ (L)	0.02 (0.18) [−0.09, −0.05]	0.536
FEV_1_/FVC (L)	0.19 (0.39) [0.031, 0.34]	0.021^1^
PEF (L/s)	−0.24 (0.85) [−0.58, 0.11]	0.173
FEF_25–75_ (L/s)	0.19 (0.55) [−0.04, 0.41]	0.096
DLCO (mL/min/mmHg)	−0.5 (3.2) [−1.9, 0.9]	0.451
DLCO/VA (mL/min/mmHg/L)	−0.2 (0.5) [−0.5, 0.0]	0.037^1^
DLCO/BSA (mL/min/mmHg/m^2^)	0.0 (1.9) [−0.9, 0.7]	0.785
DLNO (mL/min/mmHg)	−7 (16) [−13, 0]	0.060
DLNO/VA (mL/min/mmHg/L)	−1.6 (2.1) [−2.5, −0.7]	0.001^1^
DLNO/BSA (mL/min/mmHg/m^2^)	−2.6 (8.6) [−6.2, 1.0]	0.146
DLNO/DLCO ratio	−0.10 (0.28) [−0.22, −0.01]	0.076
DmCO/Vc ratio	−0.26 (0.44) [−0.45, −0.08]	0.008^1^
DmCO (mL/min/mmHg)	−22 (39) [−39, −5]	0.01^1^
Vc (mL)	0 (10) [−4, 4]	0.901

General spirometry parameters (FVC, FEV_1_, FEV_1_/FVC, PEF, FEF_25–75_, *n *=**26. Diffusing capacity parameters (Vc, DLCO, DLNO, DmCO, and all related variables), *n *=**24. The postrace measurements were obtained 73 (SD 27) minutes after marathon completion.

^1^ Indicates significant difference (*P *<**0.05) prerace to post race.

The increase in heart rate from pre to post finish was also not related to the increase in the edema score. There was no change in the proportion of individuals with clinical signs and symptoms suggestive of edema from pre to post finish with the exception of a small increase in the dyspnea score post exercise (Table 5). Arterial oxyhemoglobin and systolic blood pressure were slightly reduced post exercise, but this reduction was not clinically meaningful.

**Table 5.** Clinical signs and symptoms of pulmonary edema as well as other parameters.

	Number of subjects preexercise that were found to have the condition (*n *=**36)	Number of subjects post marathon that were found to have the condition (*n *=**36)
Bilateral crackles (end‐inspiratory crackles)	0	0
Cough, productive of frothy, or blood‐tinged sputum	0	0
Third heart sound (S_3_ gallop rhythm)	0	0

	Mean (SD) Preexercise	Mean (SD) Post exercise
Arterial oxyhemoglobin saturation via pulse oximetry (%) (*n *=**31)	98.2 (0.8)	97.7 (1.2)^1^
Heart rate (beats/min) (*n *=**33)	60 (9)	94 (12)^1^
Systolic blood pressure (mmHg) (*n *=**33)	129 (10)	120 (10)^1^
Diastolic blood pressure (mmHg) (*n *=**33)	77 (7)	76 (8)
Modified Borg scale for perceived dyspnea (*n *=**36)	0 (0)	1 (1)^1^

The modified Borg scale of perceived dyspnea (0 = nothing at all, 2 = slight, 4 = somewhat severe, 6 = very severe, 10 = the most severe out of breath you could ever imagine). The postrace measurements were taken 16 (SD 7) minutes after marathon completion.

^1^ Postmarathon values are significantly different compared to premarathon (*P *<**0.05).

Binary logistic regression revealed that there were no significant predictors of lung edema. Neither age, sex nor marathon time could predict edema (a postexercise edema score equal to or greater than 2.1 units).

## Discussion

This study confirmed previous findings (Zavorsky et al. 2014) in that some subjects were found to have mild interstitial pulmonary edema triggered by marathon running which was independent of finishing time or age. We, however, did not demonstrate that women had a higher likelihood of developing mild interstitial pulmonary edema, which is in opposition to previous findings (Zavorsky et al. 2014). What made this study novel is that we were able to collect clinical data to match the quantitative radiographic readings. There were no meaningful changes in the signs and symptoms of pulmonary edema from pre to post race despite increases in edema scores. Furthermore, while FVC, DLNO/VA, DmCO, and the DmCO/Vc ratio were statistically lower post finish, these changes pre to post exercise were not related to changes in the edema scores. This suggests a few things: First, pulmonary function cannot predict the occurrence or severity of edema; second, there is no clinical manifestation of pulmonary edema triggered from marathon running; third, the decrease in DmCO and the DmCO/Vc ratio without any change in Vc or DLCO suggests that the alveolar‐capillary membrane was thickened by mild amounts of extravascular fluid; fourth, while mild acute interstitial pulmonary edema can be detected by radiographic findings, the level of pulmonary extravascular water accumulation is so minor that clinical symptoms are not expressed post finish; and finally, the physiological significance of this finding is probably not meaningful since marathon performance was unaffected, exercise‐induced arterial hypoxemia was unlikely, and changes in pulmonary function were mild.

Not all individuals show radiographic signs of edema. In this study, about 80% did not have any edema (as evidenced by postedema score of ≤2.0 units) and neither age, sex nor marathon time could predict edema. In contrast, our previous study demonstrated that 54% did not have any edema post marathon, but being female was the best predictor of edema (Zavorsky et al. 2014). While pulmonary edema is not related to age or finishing time in this study or in the previous study (Zavorsky et al. 2014), it is probable that there are genetic (Eaton et al. 2004; Baker et al. 2011) and environmental factors that influence intersubject and interevent variability between studies.

As mentioned in our previous study (Zavorsky et al. 2014), the use of chest radiographs to detect and quantify pulmonary edema can be a limitation. However, a 1984 National Institutes of Health workshop on detection of lung water stated that *“*The chest x‐ray film remains the reference standard against which other lung water methods are compared”. Its advantages include moderate accuracy, fair sensitivity, good reproducibility, noninvasiveness, practicality, availability, reliability, portability, ease of use in the emergency care setting, and relatively low cost. It also provides excellent information about edema distribution (Staub 1986). In fact, 69% of the variance in the graded X‐ray scores for edema is accounted for by differences in the edema quantified by the indicator dilution technique (*P* < 0.01) (Pistolesi and Guintini 1978). An increase of only 10% in extravascular lung water is detectable with a chest radiograph (Pistolesi et al. 1985). The chest radiograph has therefore been rigorously confirmed as being an excellent way to detect pulmonary edema (Milne and Pistolesi 1993). Since that workshop in 1984, there has not been significant changes in the methodology for the assessment of lung edema for clinical purposes (Ware and Matthay 2005), and thus we submit that the radiograph remains an appropriate methodological technique for quantifying lung edema.

Another possible limitation is the varied edema scores between readers. We had two readers (one chest radiologist and one pulmonologist) read the films in a blinded fashion, separately, and both of them showed significant increases in the edema scores from pre to post exercise. These readers demonstrated that the same six subjects had definite increases in the lung edema score. The overall weighted kappa for all 36 subjects was 0.57, so the agreement between readers was moderate.

A further limitation was that the quality of some of the postexercise films was questionable as discussed by the initial two readers. Thus, two extra readers (J.P.R, radiologist; M.P., pulmonologist), blinded as to when the X‐rays were taken, were added post hoc to analyze the films to confirm the findings. The addition of these extra two readers showed the same mean change in the edema scores as the original two readers. Thus, the overall results were maintained. There was still an approximate threefold increase in the edema score (pre‐to‐post increase was +1.3 units) from these two different readers (on average) and the weighted kappa statistic was not different among all the four readers. So, we believe the findings, on the whole, were valid. Furthermore, five runners participated in both research studies [i.e., the 2011 Steamtown Marathon study that was published in 2014 (Zavorsky et al. 2014), and the 2012 Steamtown Marathon, which is this study]. The mean increase in the edema score in the first study for those same five subjects was +1.7, and for this current study, it was +1.0. No difference statistically (*P = *0.13), so the quality of the films for this study was sufficient.

Indeed, it could also be said that evaluating the pre‐ and postimages concurrently within a given subject (albeit blinded as to the measurement time point) was not sufficient to blind the readers. Instead, the radiographs could have been read in a randomized order from all subjects. While this has been done before in one of our previous studies (Zavorsky et al. 2006), it has been argued that this may not be appropriate because in a real‐world setting radiologists frequently views different sets of images together to see improvement or regression of a condition in a patient (Zavorsky et al. 2006). As such, we have opted for the more “real world” approach.

Furthermore, the timing of the postexercise images occurred 52 min before the measurement of pulmonary function. We had only one pulmonary function unit and we consider this to be another limitation to this study. However, radiographic findings of mild interstitial edema persist even at 98 min post marathon finish (Zavorsky et al. 2014), so we consider the time discrepancy between imaging and pulmonary function assessment to be minor.

Another potential limitation of the study is not evaluating cardiac biomarkers during exercise. A study by Favio and colleagues suggested that the increase in serum atrial natriuretic peptide (ANP) and Brain natriuretic peptide (BNP) seen after strenuous physical exercise might be associated with cytoprotective and growth regulatory effects (Faviou et al. 2008). While in the clinical world, elevations of these biomarkers are quite specific for cardiac dysfunction and congestive heart failure, it remains unclear if this explanation is valid in well‐trained athletes.

In conclusion, this study confirms that mild pulmonary edema occurs in at least 17% of subjects and that changes in pulmonary function cannot predict the occurrence or severity of edema. Furthermore, mild lung edema is of minimal physiological significance as marathon performance is unaffected, exercise‐induced arterial hypoxemia is unlikely, and postexercise pulmonary function changes are mild.

## Acknowledgments

This manuscript is dedicated to Eric Nightingale Campbell Milne M.B., Ch.B, FRCR, who passed away on December 16th, 2013. http://www.ilasting.com/ericncmilne.php. There is also a short Memoriam of him written in the May, 2014 issue of the Journal of Thoracic Imaging written by John H.M. Austin (Volume 29, Issue 3, pages W17–W18). As well, there will be another obituary of Eric Milne written by M. Pistolesi that will be published in the July, 2014 issue of Radiology. The authors would like to thank the Steamtown Marathon Race Committee, including Assistant Race Director Jim Cummings, for help in recruiting participants for this study. Additionally, we acknowledge the Steamtown Marathon Medical Director, Tim Rowland, MD, for providing available space at the medical triage area at the finish line. We also would like to thank Kathleen Uhranowsky, Kaleen Lavin, and Allison Straub for their help with data collection.

This study, in part, was presented at the American Thoracic Society Conference in Philadelphia, PA, in May 2013. The citations for both abstracts are: Zavorsky et al. (2013) and Lavin et al. (2013).

The results of this study do not constitute endorsement by the American Physiological Society or The Physiological Society.

## Conflict of Interest

All authors of this study have no conflicts of interest pertaining to this study.
